# Identifying Residual Foci of *Plasmodium falciparum* Infections for Malaria Elimination: The Urban Context of Khartoum, Sudan

**DOI:** 10.1371/journal.pone.0016948

**Published:** 2011-02-23

**Authors:** Amal B. Nourein, Mohammed A. Abass, Abdel Hameed D. Nugud, Ibrahim El Hassan, Robert W. Snow, Abdisalan M. Noor

**Affiliations:** 1 Department of Parasitology, Institute of Endemic Diseases, University of Khartoum, Khartoum, Sudan; 2 Khartoum Malaria Free Initiative, Khartoum State Malaria Control Programme, Khartoum, Sudan; 3 National Health Laboratory, Ministry of Health, Khartoum, Sudan; 4 Faculty of Medicine, University of Jazan, Jazan, Kingdom of Saudi Arabia; 5 Malaria Public Health and Epidemiology Group, Centre for Geographic Medicine, KEMRI – University of Oxford - Wellcome Trust Research Programme, Nairobi, Kenya; 6 Centre for Tropical Medicine, Nuffield Department of Clinical Medicine, University of Oxford, Oxford, United Kingdom; Université Pierre et Marie Curie, France

## Abstract

**Background:**

Identifying the location and size of residual foci of infections is critical where malaria elimination is the primary goal. Here the spatial heterogeneity of *Plasmodium falciparum* infections within the urban extent of Khartoum state in Sudan is investigated using data from cross-sectional surveys undertaken from 1999 to 2008 to inform the Khartoum Malaria Free Initiative (KMFI).

**Methods:**

From 1999–2008 the KMFI undertook cross-sectional surveys of 256 clusters across 203 random samples of residential blocks in the urban Khartoum state in September of each year. Within sampled blocks, at least five persons, including at least one child under the age of five years, were selected from each household. Blood smears were collected from the sampled individuals to examine the presence of *P. falciparum* parasites. Residential blocks were mapped. Data were analysed for spatial clustering using the Bernoulli model and the significance of clusters were tested using the Kulldorff scan statistic.

**Results:**

A total of 128,510 malaria slide examinations were undertaken during the study period. In 1999, overall prevalence was 2.5%, rising to 3.2% in 2000 and consistently staying below 1% in subsequent years. From 2006, over 90% of all surveyed clusters reported no infections. Spatial clustering of infections was present in each year but not statistically significant in the years 2001, 2002, 2004 and 2008. Spatial clusters of high infection were often located at the junction of the Blue and White Niles.

**Conclusion:**

Persisting foci of malaria infection in Khartoum are likely to distort wide area assessments and disproportionately affect future transmission within the city limits. Improved investments in surveillance that combines both passive and active case detection linked to a geographic information system and a more detailed analysis of the location and stability of foci should be undertaken to facilitate and track malaria elimination in the state of Khartoum.

## Introduction

Spatial heterogeneity in risk of malaria infection is regarded as a significant driver of the basic reproduction rate of transmission in any endemic area but becomes increasingly important as the overall intensity of transmission declines [1]–[4]. Understanding the micro-epidemiology of risk is central to adaption of wider geographical intervention policies [5]–[8] and to attempts to tackle residual foci of infections during elimination [4], [8]–[10]. How foci are indentified, their scope and scale and how one might target interventions remains poorly defined [9].

Techniques that detect the presence of statistically significant small-area clusters are often used to assess local heterogeneity of disease [6]–[8], [11]–[13]. The spatial and/or temporal clustering of malaria using these techniques have been described mainly in moderate to high transmission settings [14]–[19]. Recent applications of clustering techniques in the Sudan [7] and coastal Kenya [8] have highlighted the significance of space-time clusters of malaria infections in areas of low malaria transmission. Here the spatial clustering of *Plasmodium falciparum* infections across 256 cross-sectional surveys undertaken from 1999 to 2009 within the urban extent of Khartoum state in Sudan is examined to inform the Khartoum Malaria Free Initiative established in 2002.

## Methods

### Study Area

**Figure 1:**
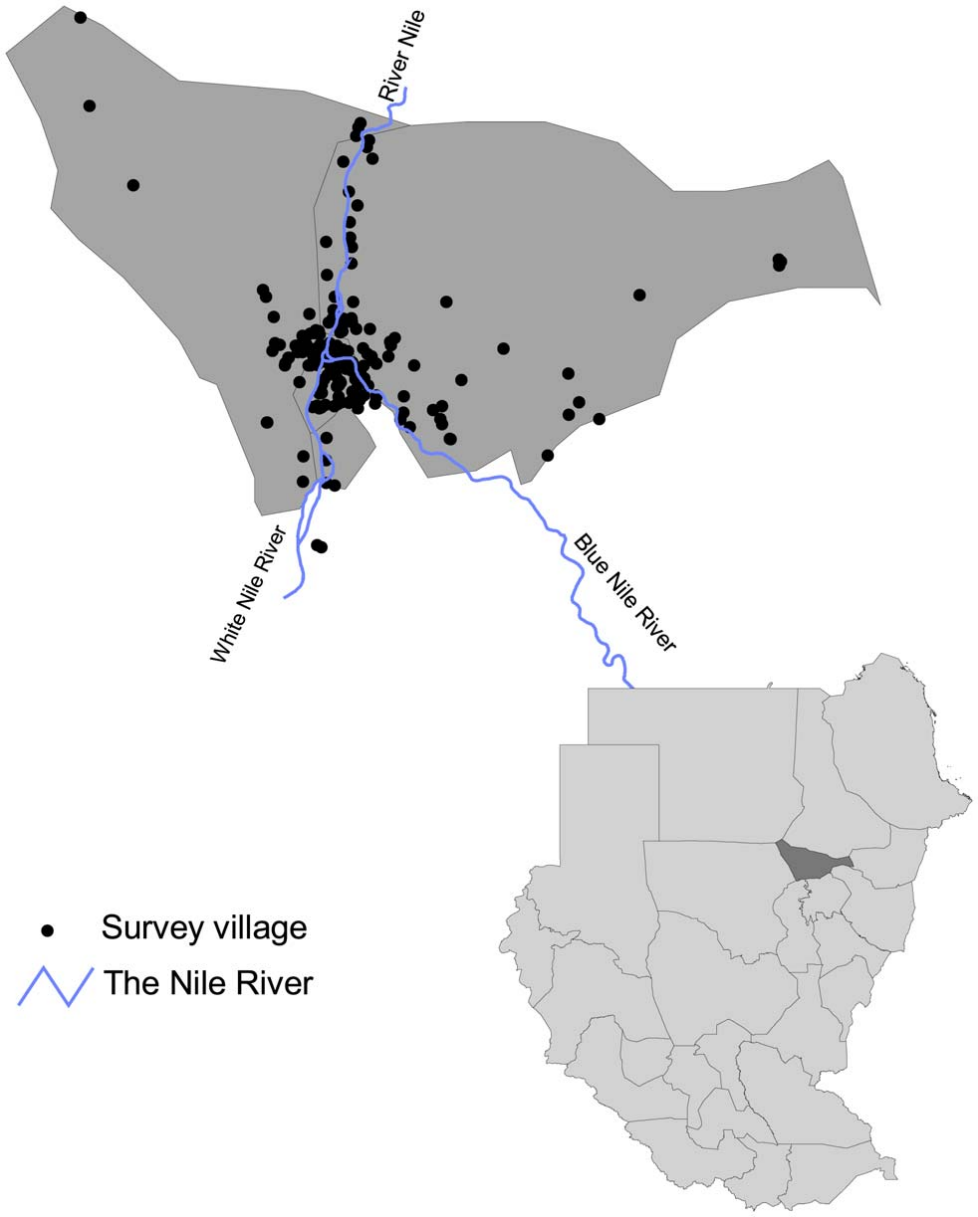
Map of Khartoum state showing the distribution of survey locations. A total of 256 *P. falciparum* prevalence surveys were undertaken in 203 locations (some survey locations were repeated in subsequent years) from 1999–2008. Inset is the state map of the Sudan showing the location of Khartoum state.

Khartoum state is one of the 26 states in Sudan with a total population of more than 5 million people in an area of approximately 28,000 Km^2^ [20], [21]. The Blue and White Nile rivers converge in Khartoum to form the River Nile along which the three administrative areas of the state: Khartoum, Khartoum Bahry and Omdurman are divided (Figure 1). The three areas differ in their malaria vulnerability due to differences in topographic, agricultural and the socioeconomic characteristics with small-scale irrigation concentrated in the Khartoum Bahry area [20]. The state experiences hot summers from April to July, patchy rains from August to October and dry winters from November to March. *Anopheles arabiensis* is the main vector of malaria [22] with the peak months of malaria transmission from September to November.

### Malaria control in Khartoum

Malaria control in Khartoum dates back to 1904 when retained oil was used as the main vector control tool leading to the eradication of the disease in the state [20], [23]. Until the 1970s, the disease incidence remained low after which the disease continued to rise until the 1990s [24]. The reduction in control efforts and increasing migration from malaria endemic states into Khartoum were thought to have contributed to this resurgence [20]. By the 1990s, malaria was a leading cause of morbidity and mortality recorded at public health facilities in the state. The federal system of governance was introduced in 1993 providing state ministries of health the power to define and implement their priority health activities. In January 1994 the Khartoum state ministry of health (KMoH) outlined plans to decrease malaria outpatient attendances by 5% every year and malaria deaths to the minimum level [24]. In 2002 the Khartoum Malaria Free Initiative (KMFI) was set up with support from the WHO and the Japanese government [20], [24]. Sustained vector control, early diagnosis, prompt treatment, and improved disease surveillance were identified as the key strategies for reducing disease burden. Microscopy was instituted in all public health facilities in Khartoum state supported with laboratory technicians trained in malaria diagnosis [24]. Hard copy maps of all breeding sites and irrigation schemes were developed and larval control using insecticides such as Temephos EC (50%) were implemented to reduce vector density. Environmental management and infrastructural improvements such as repairs of water pipes was also undertaken. Routine health facility surveillance was improved, and augmented with household and entomological surveys from 1995. A detailed description of the KMFI is provided by Khalifa et al (2008) [20].

### Parasitological surveys

Since 1995, the KMFI, led by the state malaria control programme, undertook cross-sectional community surveys during the peak transmission month of September in selected areas in Khartoum. Sample sizes were calculated on an expected prevalence of 10% which was continually revised downwards each year as prevalence decreased and, by 2004, surveys were powered on a prevalence of 0.5% [20]. Sampling was done proportionally for each of the three main areas of Khartoum state, initially starting around the junction of the Blue and White Nile rivers and expanding eventually to other parts of the state, mainly along the rivers. Communities or residential blocks were randomly selected and all households within the selected block were included in the survey. At least five persons, including at least one child under the age of five years representing the most vulnerable group due to reduced likelihood of functional immunity, were selected from each household. Thick and thin blood smears were collected from the sampled individuals. The blood films were stained with a 5% Giemsa for 30 minutes and 100 fields were examined under a 10×100 magnification microscope. A team of senior technicians rechecked at least 10% of randomly selected slides [20]. Each year a new sample was selected with previous residential blocks included in the sampling frame resulting in a random sample that contained both new blocks and some of those that were surveyed during the previous year. All individuals who tested positive for infection were treated with recommended antimalarials which was chloroquine (CQ) or sulphadoxine-pyrimethamine (SP) prior to 2004 and artesunate-SP (AS+SP) since 2004. For this study, data from 1999–2008 were used because data before this period were not available at residential level. In 2009 global positioning systems (Etrex H, Garmin Inc., USA) were used to provide a longitude and latitude for each residential block sampled since 1999.

### Spatial cluster analysis

The Kulldorff spatial scan statistic [12], as implemented in SaTScan 8.0 [25], was used for the analysis of the spatial clustering of the data, with the specific aim of identifying clusters of high *P. falciparum* infection rates. A spatial only cluster analysis was undertaken because the cross-sectional surveys relied on a fresh sample each year and consequently few clusters were surveyed continuously throughout the study period. A Bernoulli model was used for the analysis of spatial clustering in the data for several reasons. First, the number of people surveyed varied by location and it was important to adjust for these sampling changes to avoid clusters that are driven by the number of people surveyed rather than the number of people who had infection. Second, this model allowed for locations that are always of high malaria prevalence relative to other locations/years to contribute to the spatial clustering, a particularly important advantage given the generally low prevalence of the survey locations throughout the study period. The Bernoulli model requires the case and control data, represented respectively by *P. falciparum* positive and negative samples, and the spatial location for each case and control for each survey year [11], [12]. A circular spatial scan window and a maximum spatial cluster size of 50% of the cases were used so that both small and large clusters could be detected. The model compares the number of observed cases and controls in a cluster to the expectation if the spatial locations of all cases were assumed to be independent of each other. Tests of statistical significance of the identified clusters were based on likelihood ratio tests, with P-values obtained by 9999 Monte Carlo replications. The main model outputs were the location and radius of clusters, the number of villages in the cluster, the number of observed and expected cases, the rate ratio defined as the ratio of observed to expected cases of *P. falciparum* infection and the P-value of the Kulldorff scan statistic.

### Ethical Approval

Ethical approval for this study was obtained from the National Research Ethics Committee of the Federal Ministry of Health. Formal permission was obtained from the Khartoum malaria control program of the State Ministry of Health. Written consent was sought from all participants and parents/guardians of young children before blood samples were collected.

## Results

### *P. falciparum* infection prevalence

**Figure 2:**
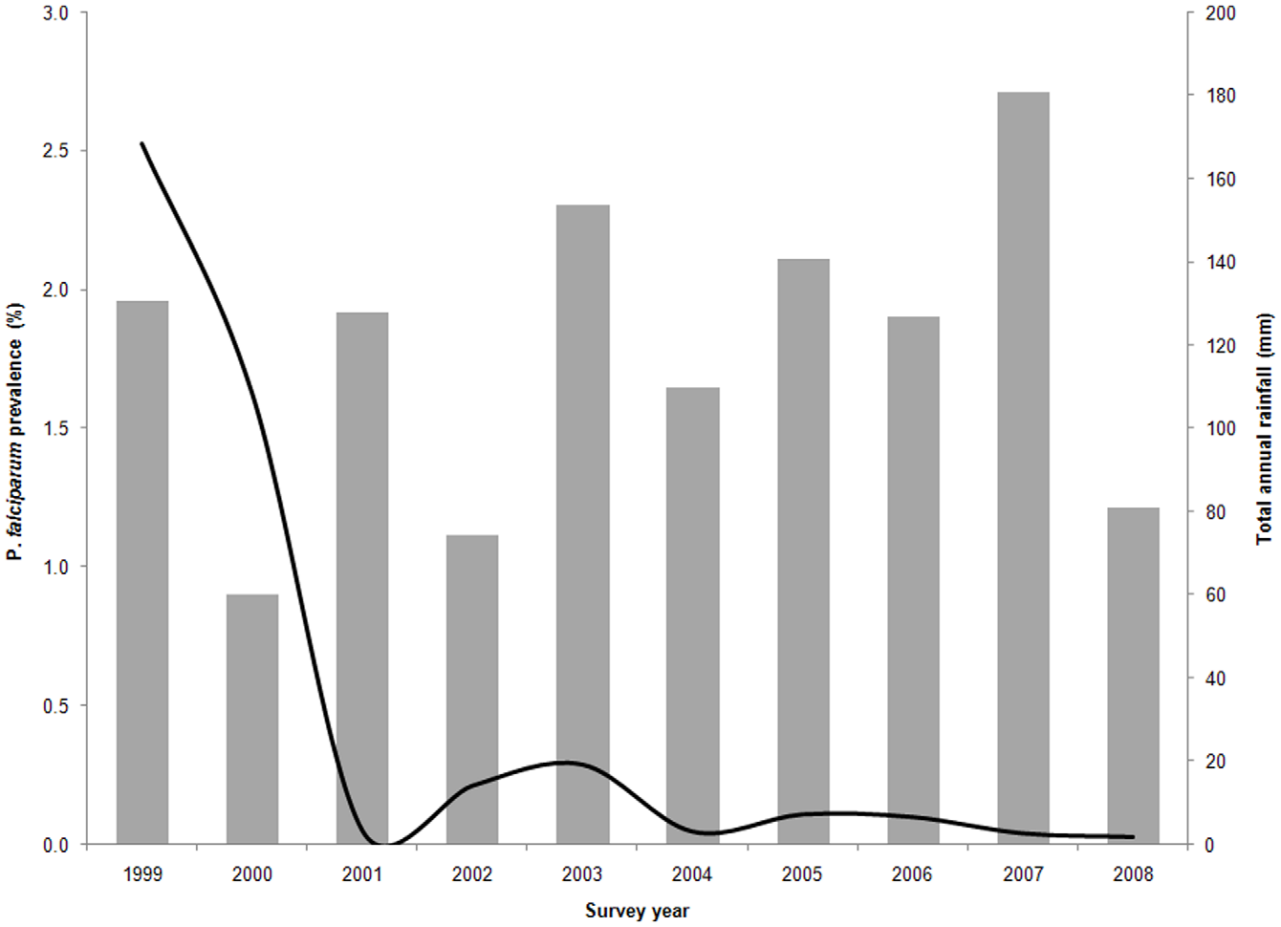
Graph of *P. falciparum* prevalence (solid line) and the total annual rainfall (bars) in Khartoum state by year of survey from 1999–2008.

From 1999–2008 a total of 128,510 malaria slide examinations from cross-sectional surveys in 256 sampled clusters in 203 residential blocks were undertaken by the Khartoum national malaria programme (Table 1). The number of individuals examined was lowest in 1999 (n = 3,561) and highest in 2003 (n = 49,180). In 1999, overall prevalence was 2.5%, rising to 3.2% in 2000 and consistently staying below 1% in subsequent years (Table 1, Figure 2). No infected individuals were found in over 70% of all clusters that were surveyed in all years after 2003. From 2006, over 90% of all surveyed clusters reported no infections (Table 1).

**Table 1:** Summary of Khartoum *P. falciparum* prevalence data from 256 surveys in 203 locations (some survey locations were repeated in subsequent years) from 1999 to 2008 showing annual average infection prevalence and the percentage and number of survey locations with no positive cases by year.

	% *P. falciparum* positive (number examined)	% survey locations (%) with no positive *P. falciparum* samples (number of survey locations)
1999	2.5 (3561)	30.0 (3)
2000	3.2 (2826)	11.1 (1)
2001	0.1 (8092)	80.0 (12)
2002	0.1 (8092)	40.0 (6)
2003	0.2 (49180)	0.0 (0)
2004	0.8 (11230)	75.0 (15)
2005	0.9 (10295)	89.2 (33)
2006	0.9 (10295)	94.6 (35)
2007	0.7 (13069)	94.6 (35)
2008	0.8 (11870)	92.5 (37)
**Total**	0.1 (128510)	69.1 (177)

### Annual spatial only clustering in *P. falciparum* infection prevalence

**Figure 3:**
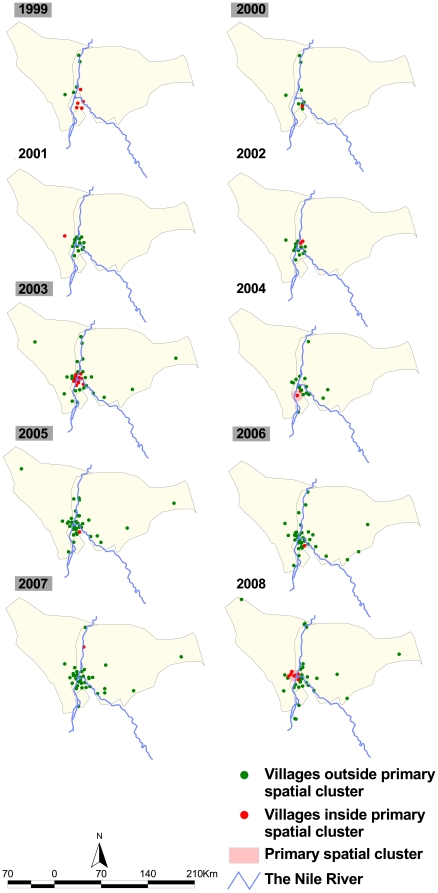
Location of primary spatial clusters of *P. falciparum* prevalence. Primary spatial clusters in Khartoum state in each year from 1999 to 2008 are shown as pink circles of varying radius or red zeros (when a cluster radius is indeterminate). The primary spatial clusters in 2001, 2002, 2004 and 2008 were all statistically not significant (Kulldorff scan statistic of >0.05). Years with statistically significant spatial clusters are shaded grey.

The Kulldorff spatial scan statistic showed spatial clustering of *P. falciparum* prevalence in each year from 1999–2008 (Table 2 and Figure 3). However, these were not significant in the years 2001, 2002, 2004 and 2008. In 2000, 2001, 2005, 2006 and 2007 the spatial clusters were of indeterminate radius i.e. only a single community was identified to be in the cluster and hence the cluster radius was 0 km (Table 2). In the other years, the number of villages within a spatial cluster ranged from one to eight and cluster radius of 4 km in 2002 to 16 km in 1999. The rate ratio of the spatial cluster defined as the risk of an individual within the cluster having an infection compared to those outside was highest in 2006 (RR = 297.9) and lowest in 1999 (RR = 2.1). In each of the study years the mean *P. falciparum* prevalence within the spatial clusters was considerably higher than that outside the clusters (Table 2) with the differences generally increasing with declining prevalence. The proportion of malaria positive cases within a cluster where infection was documented ranged from 17% in 2000 to 67% in 2008. In all the years where significant spatial clusters of high infection rates were observed, these were located around the junction of the Blue and White Niles (Figure 3) and were qualitatively proximal to agricultural sites.

**Table 2:** Primary spatial only *P. falciparum* clusters, their radius, the number of survey locations and cases contained in the clusters and the significance of the Kulldorff scan statistic in Khartoum state from 1999 to 2008.

Year	Number of clusters	Number of Villages in cluster	Radius of cluster (Km)	% Examined inside cluster	% *P. falciparum* cases inside cluster	Relative Risk	P-Value	% *P. falciparum* positive inside cluster	% *P. falciparum* positive outside cluster
1999	1	4	15.8	43.7	62.2	2.1	0.0048	3.3	2.0
2000	1	1	0.0	5.3	17.4	3.8	0.0262	5.3	1.4
2001	1	1	0.0	7.1	50.0	13.1	0.4881	0.4	0.0
2002	1	2	4.0	12.9	41.2	4.7	0.0746	0.7	0.1
2003	1	9	9.9	26.6	45.4	2.3	0.0005	0.5	0.2
2004	1	3	7.9	17.4	60.0	7.1	0.5668	0.2	0.0
2005	1	1	0.0	0.8	63.6	223.5	0.0001	8.8	0.0
2006	1	1	0.0	0.8	70.0	297.9	0.0001	8.8	0.0
2007	1	1	0.0	1.3	60.0	110.5	0.0008	1.7	0.0
2008	1	8	7.9	19.0	66.7	8.5	0.7658	0.1	0.0

## Discussion

The analysis of data assembled from 256 community cross-sectional cluster surveys of *P. falciparum* infections in 203 residential blocks from 1999 to 2008 in the state of Khartoum show overall low prevalence beginning at 2.5% at the start of the surveillance period and declining four-fold to 0.8% by 2008. This overall decline masks the presence of primary spatial clusters in all years, with these being statistically significant in six of the ten years of observation. The mean prevalence of infection in residential blocks contained in the primary spatial clusters was consistently higher than that of the overall data throughout the study period. The location of the statistically significant primarily clusters were mainly around the junction of the Blue and White Nile rivers.

A review of the KMFI activities by Khalifa et al. (2008) [20] showed that the initiative had achieved significant progress in malaria diagnosis and case management, mapping of vector breeding sites and insecticide control and improvement in health information systems in Khartoum. In addition, the federal National Malaria Control Programme (NMCP) of the northern states of the Sudan has distributed almost 0.6 million long lasting insecticidal nets (LLINs) in the state since 2006 [26]. Although it is not possible from the available data to directly attribute the exact impact of these interventions on malaria infection prevalence there is a strong coincidence with the expansion of the KMFI activities [20] and the observed reductions in prevalence are unlikely to be associated with changing patterns of rainfall (Figure 2). A recent national malaria indicator survey undertaken in October-November 2009 sampled 3,214 individuals located in Khartoum who were tested with rapid diagnostic test and showed a *P. falciparum* prevalence 0.1% supporting the findings presented here of extremely low prevalence in the state of Khartoum in 2009 [26]. The analysis of spatial clustering of malaria infections, however, reveals the consistent presence of clusters of high rates at the confluence of the Blue and White rivers. This is a mainly agricultural area with many fruit and vegetable farms including those located at Tuti, the island between the Blue and White Nile rivers at the point where they converge.

The analysis here demonstrates the utility of spatial cluster analysis techniques to help identify possible residual foci of infections in an area of very low malaria transmission. The observed heterogeneity of risk therefore implies that clusters of high prevalence contribute disproportionately to wide area mean estimates of infection prevalence and is likely to be the main reservoirs of continued transmission [3]. These foci described in Khartoum appear to be connected with the areas where agricultural activities are more common and where the potential for mosquito breeding is likely to be maintained, an observation recently made further south at Gezira state [7]. The present study, however, had several limitations. First, only a few residential blocks were surveyed continuously throughout the study period and it was only possible to employ a spatial-only cluster analysis without a temporal dimension. Future data collection should explore the possibility of long term repeat surveys in sample communities. Second, while the KMFI has invested heavily in control interventions their distribution and coverage are not available within a compatible spatial platform to examine attribution. Third, questions on travel history were not asked during household surveys and this limits the possibility of distinguishing locally acquired infections from those that are imported. This is particularly important in the study of spatial clustering in low transmission, urban settings where in-migrants might concentrate in volume and origin within defined areas [27]. Finally, as infection prevalence continues to decline microscopy or rapid diagnostic tests will have low detection rates [28] and could underestimate the overall infection prevalence. Alternative approaches such as of polymerase chain reaction to detect low level infections should be explored.

Furthermore, although only one case of *P. vivax* cases was observed across the study period [unpublished data] there is need for a much better understanding of the burden of this parasite. This is particularly important given the challenges to elimination posed by the hypnozoite, liver stage of the parasite life cycle, which stays dormant for long periods and a single infection can lead to a series of relapses [9], [29]–[31].

Surveys of residential blocks and enhanced clinical surveillance should be improved to define progress and possible success in malaria elimination. The declining levels of infection prevalence will render community cross-sectional parasite surveys inefficient in monitoring transmission over time and expanding them to the required sample sizes will become very expensive [4], [32]. Parasitologically confirmed clinical cases of malaria should be investigated for travel histories [33] and linked to residential maps [9], [34], [35] that should serve to signal investigations of all secondary cases and a survey of surrounding residents for parasite carriage [36]. These systems take time to establish and the cluster analysis presented in this paper of residual foci of infection may serve to focus initial investments in sentinel passive-active clinical detection systems. However, although the KMFI has scaled up parasitological diagnosis of malaria to all public health facilities in Khartoum, the health facility information system remains inefficient and cross-sectional community surveys and entomological studies were last undertaken in 2008 due to shortage of funding. This has the potential of reversing the impressive gains made so far by the KMFI.

This study demonstrates the potential of cluster analysis techniques in identifying the location and radius of spatial clusters of malaria infection which are likely to be residual foci of infections. The results provide evidence that the KMFI has reached a level of measurable success however the remaining foci are likely to distort wide area assessments and disproportionately affect future transmission within the city limits. Data of higher spatial and temporal resolution with detailed series of individual, household and cluster level predictors of infections are required to for a comprehensive assessment of the location and stability of residual foci of infections in Khartoum state. Bespoke versions of the cluster analysis techniques could be developed for applications to new forms of clinical case detection data with sufficient investment in health information systems.
